# Flow-Based Single Cell Deposition for High-Throughput Screening of Protein Libraries

**DOI:** 10.1371/journal.pone.0140730

**Published:** 2015-11-04

**Authors:** Cassandra Stowe, Arnold Pizzey, Tammy Kalber, Adam Badar, Mark Lythgoe, Martin Pule

**Affiliations:** 1 Cancer Institute, Department of Haematology, Division of Medicine, University College London, London, United Kingdom; 2 Centre for Advanced Biomedical Imaging, Division of Medicine, University College London, London, United Kingdom; University of Houston, UNITED STATES

## Abstract

The identification and engineering of proteins having refined or novel characteristics is an important area of research in many scientific fields. Protein modelling has enabled the rational design of unique proteins, but high-throughput screening of large libraries is still required to identify proteins with potentially valuable properties. Here we report on the development and evaluation of a novel fluorescent activated cell sorting based screening platform. Single bacterial cells, expressing a protein library to be screened, are electronically sorted and deposited onto plates containing solid nutrient growth media in a dense matrix format of between 44 and 195 colonies/cm^2^. We show that this matrix format is readily applicable to machine interrogation (<30 seconds per plate) and subsequent bioinformatic analysis (~60 seconds per plate) thus enabling the high-throughput screening of the protein library. We evaluate this platform and show that bacteria containing a bioluminescent protein can be spectrally analysed using an optical imager, and a rare clone (0.5% population) can successfully be identified, picked and further characterised. To further enhance this screening platform, we have developed a prototype electronic sort stream multiplexer, that when integrated into a commercial flow cytometric sorter, increases the rate of colony deposition by 89.2% to 24 colonies per second. We believe that the screening platform described here is potentially the foundation of a new generation of high-throughput screening technologies for proteins.

## Introduction

The identification and isolation of proteins having refined or novel characteristics is an important area of research in many scientific fields, examples include therapeutics[1], antibody production[2] and various imaging modalities[3,4]. In recent years, advances in high-throughput protein modelling and screening techniques have provided researchers with powerful tools, facilitating the rational design of proteins with enhanced or *de novo* characteristics [5]. However, despite these advances, the rational design of proteins remains a formidable challenge due to the large combinatorial space to be explored and thus, there remains a need for new high- throughput screening strategies[6].

The screening of large protein libraries for specific binding characteristics is an area where mass screening techniques such as phage display have proved of enormous facility[7]; and proteins that express a stable fluorescent property may be rapidly screened and selected based on this property by for example, Fluorescence Activated Cell Sorting (FACS)[8]. There remains however, groups of proteins which have proven refractory to the above approaches such as small molecule binding proteins[9] and enzymes[10]. In addition, proteins used in imaging technologies such as bioluminescent proteins[11,12] for optical imaging or complex fluorescent proteins[13,14] for super-high resolution microscopy (PALM[15]/STORM[16]) are also difficult to screen in a high-throughput manner. In these cases, the requirement for single cell analysis means liquid handling robots are of limited facility so current screening methods are largely restricted to traditional, microbiological techniques which are poorly matched with the more recent advances in genetic biology, which typically produce very large libraries of mutant proteins[17,18].

Using a combination of classical microbial culture approaches along with FACS techniques, we address the problem of the large scale screening of proteins with no binding capacity or stable fluorescent marker. Here we report the development of a novel FACS-based screening platform. Single bacterial cells, expressing a protein library to be screened, are electronically sorted and deposited onto solid nutrient growth media in a dense matrix format. We show this matrix format is readily applicable to high-throughput machine interrogation and interpretation, thus enabling the large scale screening of the protein library. To further enhance this screening platform, we have developed an electronic sort stream multiplexer, that when integrated into a commercial flow cytometric sorter, increases the speed of colony deposition by almost 10-fold.

## Methods and Results

### Development of FACS single colony deposition

**Fig 1:**
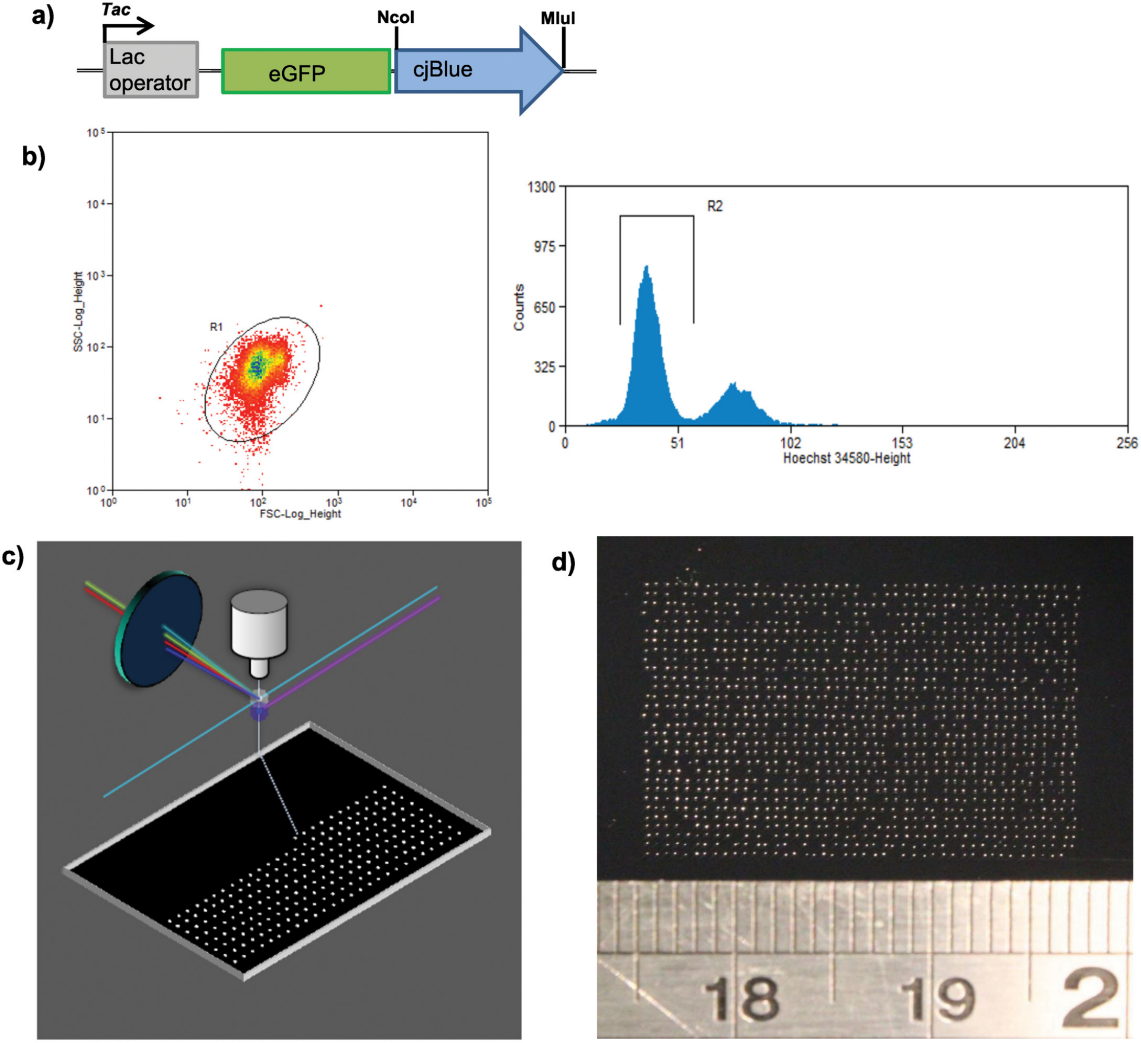
Workflow for single colony deposition. a) The open reading frame of the bacterial screening vector, showing the fluorescent protein marker eGFP in frame with the stuffer sequence cjBlue. cjBlue is flanked by the universal restriction sites NcoI/MluI to enable the insertion of protein libraries to be screened using the platform. b) FACS data obtained from bacteria transformed with the screening vector. The DNA binding dye Hoechst 34580 was used to identify high viability bacterial cells (Gate R2). Data within the R2 gate was further analysed for forward vs side scatter to identify noise events which were excluded using gate R1. Note that bacteria excluded from gate R2 are not non-viable. In initial experiments when a gate was placed over the higher intensity Hoechst peak there was a reduction in printed bacterial viability of 10% (n = 6’600) compared to the lower intensity R2 gated Hoechst peak. c) FACS single bacterial cell matrix deposition. Those bacteria falling within the electronic gates R2, R1 and displaying eGFP positivity were identified as candidates for single-cell deposition and were deposited in a matrix-format onto black charcoal agar plates for subsequent overnight incubation and analysis. d) Insert showing ultra-high-density bacterial deposition obtained from the workflow. Approximately 1,300 bacterial colonies have been deposited in a 22*15mm area (~394 colonies/cm^2^) with colony spacing of ~500μm.

The bacterial expression plasmid pGex-6p-2 was engineered to be a vector for the inducible expression of proteins to be screened using this platform. The enhanced green fluorescent protein (eGFP) was included as a marker for FACS analysis. Inserted in frame downstream by a flexible serine/glycine linker is the stuffer complex cjBlue flanked by the universal restriction sites NcoI/MluI for easy replacement by any protein to be screened (Fig 1a). Bacterial suspensions were prepared by transforming the screening vector into competent *E*.*Coli*, which were subsequently expanded in nutrient growth media. Protein production was induced by addition of IPTG and bacteria were stained with the fluorescent DNA-binding dye Hoechst 34580 prior to FACS analysis.

Briefly, Hoechst 34580 fluorescence was selected as the primary sort determinate; and further electronic gates were applied to the data, selecting for bacteria having light scatter properties and Hoechst 34580 staining which correlate with viable cells (Fig 1b). In some experiments, additional gating strategies were employed; utilizing fluorescent protein expression to ensure successful bacterial transformation. In contrast to standard bulk sorting modalities, we adopted a single-cell approach where single bacterial cells meeting all relevant sort criteria were deposited in a dense matrix onto the surface of solid bacterial growth medium (Fig 1c). A charcoal containing solid nutrient medium was used in all single cell depositions to facilitate a low background for subsequent optical imaging. After overnight incubation, plates were sprayed with highly aerosolised IPTG to induce protein production in colonies before being analysed.

In the development phase of the technique, single bacteria were printed in a 384-point format (16*24). All subsequent higher density printing kept this 1:1.5 ratio of rows to columns to fit the aspect ratio of commonly available micro-well format plates. High density plates were further sub formatted into blocks to ensure easy selection of colonies subsequently identified as containing a protein of interest. The highest density plate printed using this method contained 25,350 bacterial colonies with approximately 500μm spacing (130*195) (Fig 1d). The average viability of deposited bacteria using this method was 94% (n = 20, SD = 4.25), so a plate printed in a 3,750 matrix would contain approximately 3,525 individual bacterial colonies. A detailed protocol for this new FACS based screening technique can be found in supporting information (S1 Protocol).

**Fig 2:**
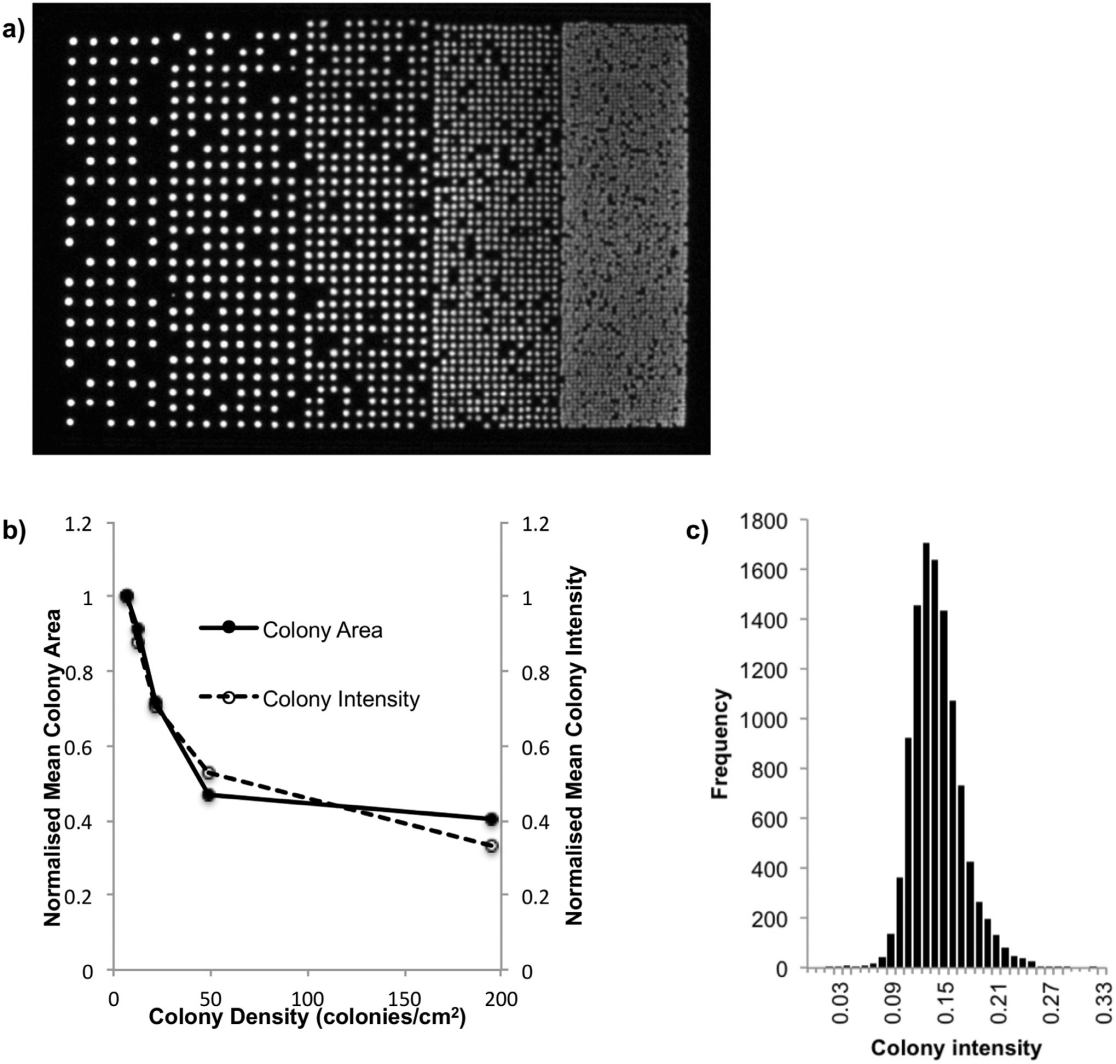
Evaluation of matrix density and colony variability. a) Individual bacteria transformed with the screening vector, containing the bioluminescent protein luciferase from the firefly *Photinus pyralis*, were printed in a matrix format at increasing density onto ablack charcoal agar plate, ranging from ~7 colonies/cm^2^ to 195 colonies/cm^2^. After overnight incubation, the plate was sprayed with highly-aerosolised D-luciferin and imaged with a sensitive image-intensified camera (PhotonIMAGER^™^Optima, *Biospace lab*).The resulting image was analysed using the CellProfiler software and intensity values, representing protein production from Firefly luciferase, and surface area measurements were obtained for each individual colony. b) Plot showing the mean individual colony area and the mean individual colony intensity for each colony at each density printed; both colony area and protein production per colony decrease with increasing matrix density. c) Frequency distribution of colony intensity values, as reported by CellProfiler, of 10’788 colonies of a clonal population of bacteria expressing Firefly luciferase after spraying with D-Luciferin.

To establish the matrix density that could be printed and analysed using this FACS based single colony deposition protocol a plate was printed with increasing matrix density, ranging from ~7 colonies/cm^2^ to 195 colonies/cm^2^ (Fig 2a). Fig 2b shows the relationship between increasing matrix density and individual colony area, as well as increasing matrix density and protein production per colony through the relative light units of bioluminescence emitted by the luciferase protein from *Photinus pyralis* (FLuc). With increasing matrix density a decrease in both single colony area and protein production per colony was shown. FLuc intensity was also used to validate the variability between colonies printed using this method, and the consistency of the spray application. A clonal population of bacteria expressing FLuc were printed, and the resulting plates were then imaged on the PhotonIMAGER^™^Optima after been sprayed with D-Luciferin. Mean intensity of 10’788 individual colonies, as analysed using CellProfiler Software, was found to be 0.14 (SD±0.03). A frequency distribution of colony intensity is shown in Fig 2c.

### Validation of single bacterial cell printing

**Fig 3:**
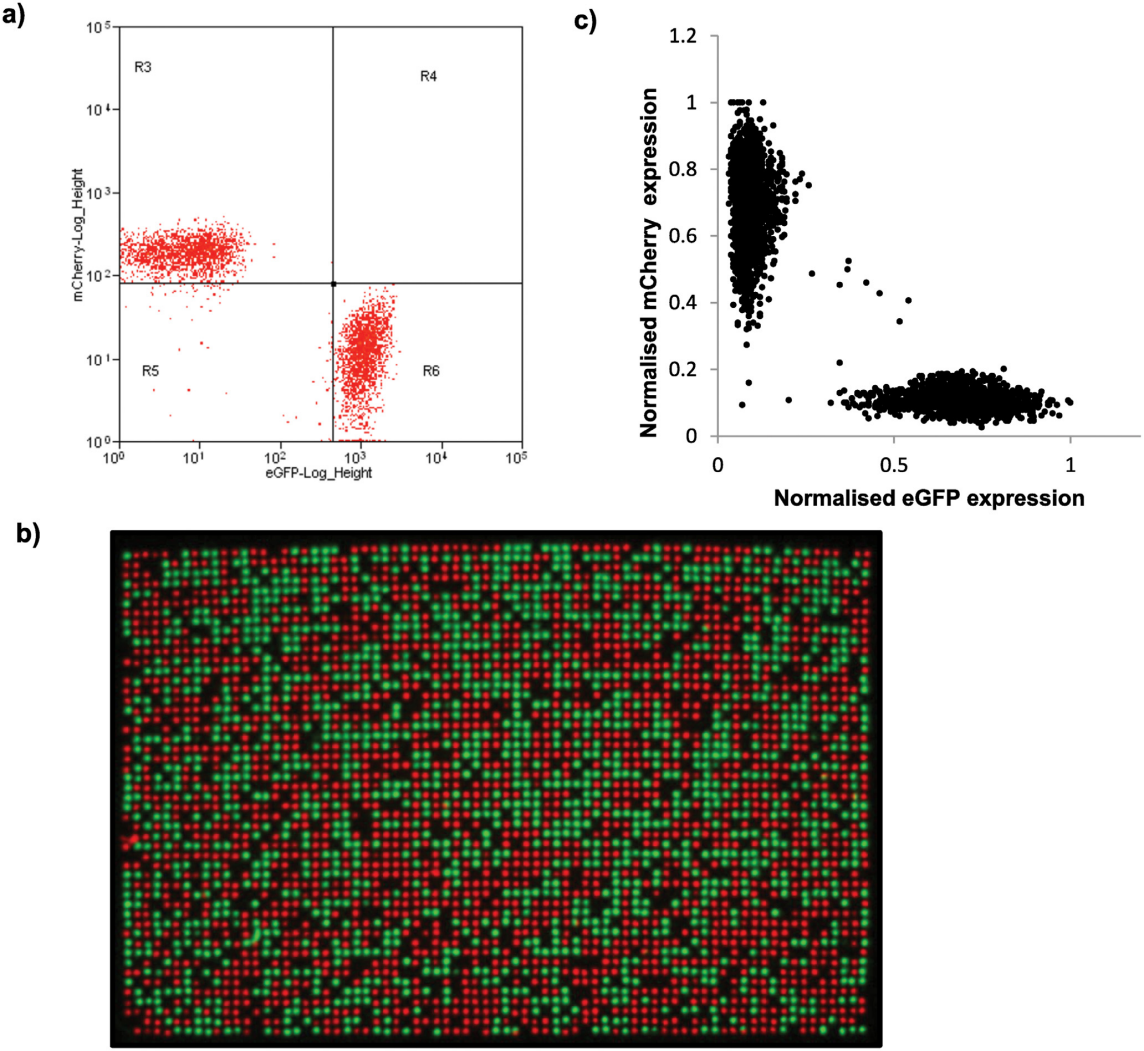
Single bacterial cell deposition and analysis. To determine whether a single colony had been deposited in each position of the matrix a mixture of bacteria expressing either mCherry or eGFP were put through the screening platform. a) FACS scatter plot showing the input population (ratio approximately 1:1 of eGFP:mCherry expressing bacteria). Bacteria were then printed by gating off Hoechst 34580 and forward vs side scatter (as shown in Fig 1b); there was therefore an equal chance of an eGFP or an mCherry expressing colony been deposited in each position of the matrix. b) After overnight incubation plates were imaged for both eGFP and mCherry expression in the PhotonIMAGER^™^Optima (*Biospace lab*). An example of an eGFP / mCherry fluorescence composite image derived from the single cell sort. The composite image was analysed using CellProfiler software. c) The resulting intensity values were then used to plot intensity of eGFP against mCherry for each of the colonies displayed in Fig 3b, highlighting any double positivedepositions. It should be noted that, the purpose of this analysis was to demonstrate coincident fluorescent events and not illustrate the relative quantification performance between the flow cytometry data (Fig 3a) and the data obtained from the optical imager shown in this plot. Even though the same fluorescent protein expressing bacteria are analysed, the two technologies differ in interrogation source and discrimination method, which therefore give data with different resolutions and signal-to-noise ratios.

To confirm single bacterial cell sorting and printing, samples of bacteria were singularly transformed with plasmids (pGex.6p-2) containing either the fluorescent protein eGFP or mCherry. After bacterial preparation as described above, a sample containing a mixture of both eGFP expressing bacteria and mCherry expressing bacteria was analysed by FACS and the composition of the mixed sample was subsequently adjusted to a 1:1 ratio of eGFP: mCherry to ensure equal probability of eGFP or mCherry expressing bacterial deposition (Fig 3a). Bacteria were then sorted onto the solid growth medium plate in a 3,750 matrix formation (50*75) by triggering only off Hoechst 34580 fluorescence.

Each bacterial colony was then assessed for eGFP and mCherry expression by acquiring fluorescent images on the PhotonIMAGER^™^Optima (*Biospace Lab)*. One image was acquired using excitation and emission settings corresponding to eGFP (488nm_ex_/510nm_em_), and another image was taken for mCherry (575nmex/610nmem). These images were then overlaid using the freeware image manipulation application Image J [19] (Fig 3b) and then further analysed using the freeware application CellProfiler[20]. The scatter plot shown in Fig 3c generated from the CellProfiler data shows the eGFP and mCherry intensity levels for each individual colony in the plate shown in Fig 3b. Out of 12,721 bacterial colonies printed over 4 plates by just triggering off Hoechst 34580 a single colony expressed only eGFP or mCherry in >99.8% of cases (SD±0.066). This shows a high level of stringency to single colony deposition, and depending on the use of the platform any false positive or negative results arising from double colony deposition would be low (<0.4%) taking in to account the inability to identify coincident same colour depositions.

### Evaluation of screening platform using bioluminescence

After validating that individual fluorescent bacterial deposition and detection had been achieved, we set out to determine whether a non-fluorescent protein could be screened using this platform. The bioluminescent protein Luciferase from the North American Firefly *Photinus pyralis* (FLuc) was chosen as a screening candidate. A variant of FLuc with 5 temperature and pH stabilising mutations, (x5_FLuc) was used with an emission maxima of ~550nm [21]; as well as a variant of x5_FLuc with a point mutation (S284T) causing red-shifted light emission to a max of ~615nm (x5_FLuc_red) [22,23]. The cjBlue stuffer sequence in the screening vector was replaced by the sequence for either x5_FLuc or x5_FLuc_red by NcoI/MluI digestion and ligation. Both constructs were singularly transformed into bacteria and prepared for FACS analysis and single bacterial cell deposition, as described above.

Bacteria were printed in a 3750 format (50*75) by triggering off Hoechst 34580 and the screening vector marker eGFP. The first 25 rows were printed with bacterial sample containing x5_FLuc, the remaining 25 rows were printed with bacteria containing x5_FLuc_red. Plates were first sprayed with highly aerosolised FLuc substrate D-Luciferin using a commercial airbrush, before been imaged for bioluminescence on the PhotonIMAGER^™^Optima. After a 120 second delay to allow for stabilisation of light output from FLuc a series of images were acquired through six optical band pass filters ranging from 510nm to 750nm, covering the emission maxima of both x5_FLuc and x5_FLuc_red. Therefore to process one plate in this way takes less than 4 minutes, and if a full, six-point spectral dataset is not required, the imaging would take less than 3 minutes. In the case of fluorescent proteins, where there is no requirement for substrate addition an image can be acquired in less than 30 seconds.

The six images for each plate were exported as a Portable Network graphic (PNG) file for subsequent analysis using the CellProfiler application. In CellProfiler, an image processing pipeline was created to be able to reliably identify individual bacterial colonies and to further quantify light emission from each colony in each filter image. To process a full set of images through CellProfiler in this way takes approximately 60 seconds (depending on the speed of the workstation used). The data obtained from CellProfiler was further analysed to produce a normalised 6-point spectrum for each colony, and any colony with a spectral shift was identified on a recreated plate map.

**Fig 4:**
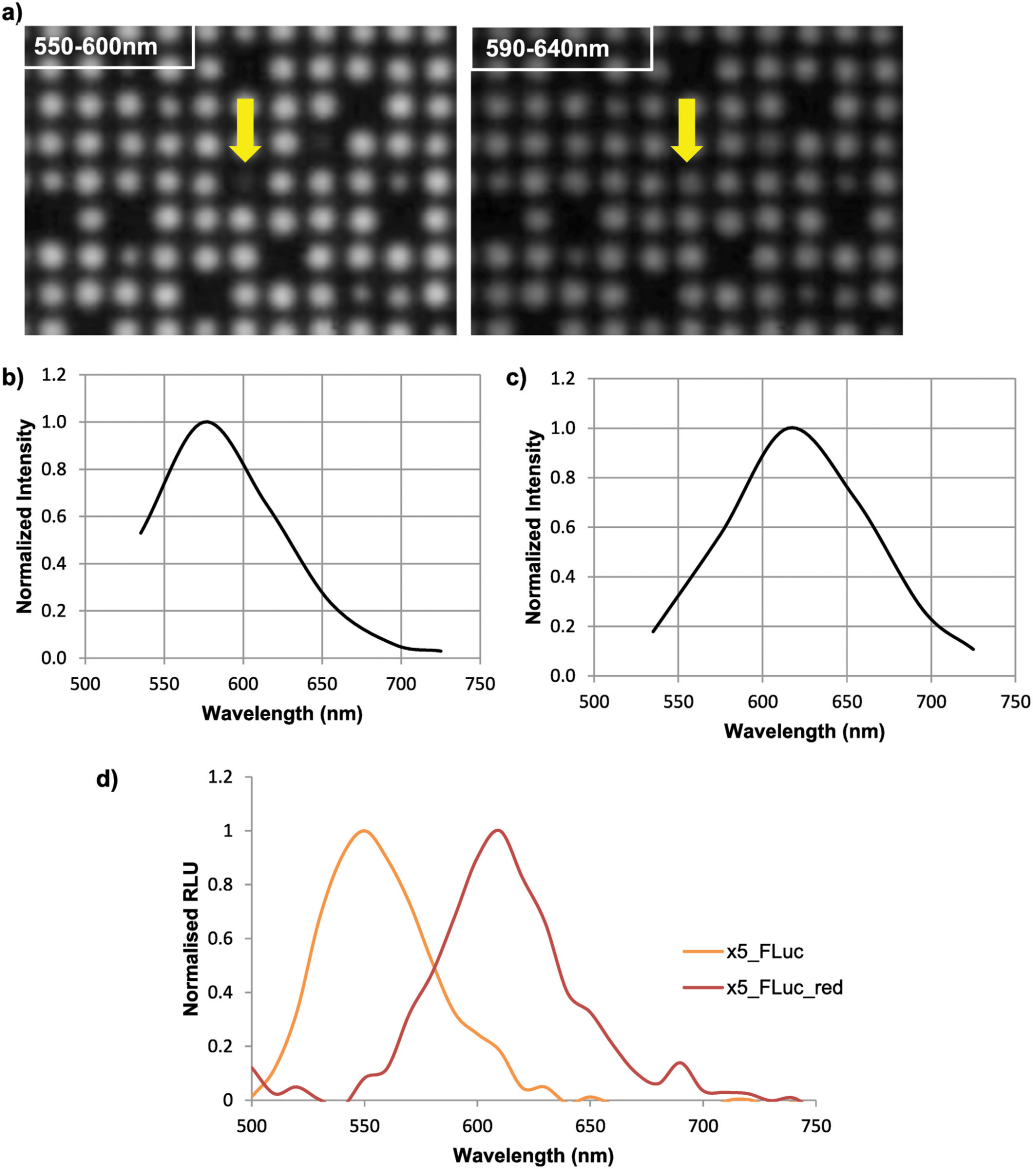
Evaluation of platform using a bioluminescent protein. Bacteria were transformed with the screening vector containing either the firefly luciferase variant x5_FLuc (~550nm) or a red-shifted variant of firefly luciferase x5_Fluc_red (~615nm). An input sample, composed of x5_FLuc expressing bacteria with x5_Fluc_red expressing bacteria spiked in at 0.5%, was printed using the screening platform. a) Bioluminescent images of the plates were then acquired using PhotonIMAGER^™^Optima (*Biospace lab*) through 6 bandpass filters ranging from 510-750nm. Images shown represent sections of a plate acquired through a 550-600nm bandpass filter and a 590-640nm bandpass filter, the same red shifted FLuc colony is highlighted in both images. Images were then analysed using CellProfiler software, giving an intensity value for each individual colony through each of the 6 filters to give a 6-point bioluminescent emission spectrum. b) Shows the spectrum for an x5_Fluc colony and c) shows the spectrum for an x5_Fluc_red colony. d) Finally, a x5_Fluc colony and a x5_FLuc_red colony were picked and a bioluminescent emission spectrum was taken using crude bacterial lysates using a multimodal plate reader (10nm intervals between 500-750nm) to confirm the bioluminescent emission spectrum produced through the screening platform.

To further support that spectral mutants of bioluminescent proteins could be reliably identified using our screening platform; a matrix plate was printed from a sample of bacteria expressing x5_FLuc that had bacteria expressing x5_FLuc_red spiked in at 0.5%. Fig 4a shows the resulting bioluminescent images of a section of this plate through two of the six filters, bandpass 550-600nm and 590-640nm; highlighting the same x5_FLuc_red colony in both images. After processing and analysis as described above using CellProfiler, a six-point emission spectrum was produced for each individual colony, identifying any containing the red-shifting FLuc mutation. An example of an emission spectrum produced for x5_FLuc and x5_FLuc_red is shown in Fig 4b and 4c respectively. Those colonies identified as expressing the x5_FLuc_red were picked for further analysis to verify the presence of the red-shifting FLuc mutation, as well as a colony identified as expressing the naturally emitting x5_FLuc. The emission spectrum of the expanded cultures from each picked colony was obtained with a spectral resolution of 10nm on a micro-well plate reader and the FLuc coding region of the screening vector for each colony was sequenced to confirm the S284T mutation. An example of the emission spectrum from x5_FLuc and x5_FLuc_red produced in the micro-well plate reader is shown in Fig 4d. Spectral and sequence data supported that the screening platform could reliably identify spectral mutants of bioluminescent proteins.

Utilising this method we have shown that a rare clone (0.5%) of a bioluminescent protein may be identified and then successfully picked and further characterised using this platform. To assess the relationship of colony density to the accuracy of subsequent picking, a mixture of bacteria expressing either eGFP, mCherry, x5_FLuc or x5_FLuc_red in a 1:1:1:1 ratio were printed at three densities; 44, 89 and 174 colonies/cm^2^with no specific gates set for any of the specified proteins. After optical imaging (as described above), 15 colonies expressing each of the proteins in the sample were manually picked at randomly selected positions from each of the three plates. The picked colonies were re-plated on to solid medium and analysed. Picking was carried out in normal laboratory lighting conditions with no additional magnification to aid the operator to give the severest test of the methodology. Out of a total of 180 picked and re-cultured colonies (n = 60 per density) there were three incidences where the target colony was not observed on subsequent reculture, these incidences only occurred at the highest density tested (174 colonies/ cm^2^); two x5 FLuc picked targets were found on subsequent image analysis to be x5_FLuc_Red, and a single x5_FLuc_Red was found to be x5_FLuc. Furthermore, there were a total of five incidences (out of the 180 colonies picked) where non-target colonies were observed in addition to the target colonies (two from 174 and three from 89 colonies/cm^2^ plate densities). Therefore, in our hands, using this manual picking method, the apparent error of picking was 3 out of 60 when colonies were printed at a density of 174 colonies/ cm^2^ (the highest density tested).

### Further development and application of screening platform

To further improve the throughput of our platform we have devised and fabricated an electronic sort multiplexer device that, when retrofitted to our electronic flow sorter speeds up the rate of single particle deposition by almost tenfold. The prototype device consists of a commercial, low-cost single-board microcontroller running our own control software linked to a fast, high-voltage programmable attenuator board, which has been designed and fabricated by us. Schematics for the multiplexing sort device can be found in Figs A & B in S1 File.

**Fig 5:**
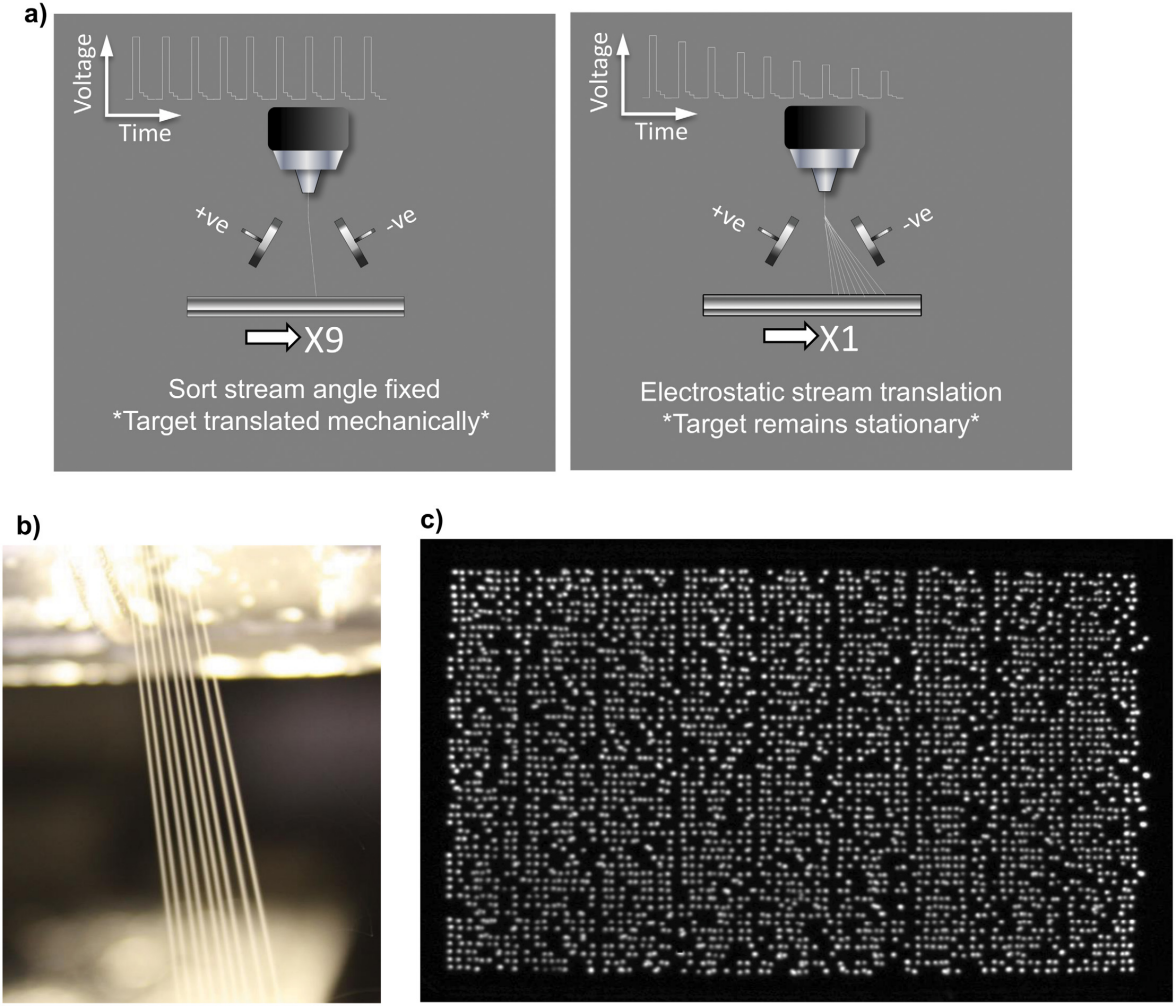
Comparison of conventional electromechanical target translation vs sort stream multiplexing. a) The conventional methodology predicates that the target is physically moved to the next position before the machine can deposit a particle which incurs a cumulative time penalty. With electrostatic stream translation, the target remains stationary during each burst. b) Image demonstrating the 9 streams produced by the electronic sorter when using the multiplexer device. c) A bioluminescent image of a bacterial plate deposited using the sort-multiplexing device. The bacteria were printed in high-speed bursts in groups of 9 to form this 4,050-point matrix.

The device intercepts the sort charge pulse train and applies an increasing attenuation function to successive pulses and, as each pulse is attenuated, the corresponding droplet/particle trajectory angle is reduced (Fig 5a). As a consequence, the device allows the machine to ‘write’ individual bacteria as single cells to the surface of the growth medium in bursts at the sort rate, of the MoFlo^™^ XDP Electronic Cell Sorter (*Beckman Coulter*) (Fig 5b). The major rate-limiting factor operating when using the electronic sorter is the ‘dead time’ during which the electromechanical plate handler is transiting from one deposition point to the next, therefore any strategy which mitigates against this rate limit has the potential to dramatically increase the speed of throughput.

**Fig 6:**
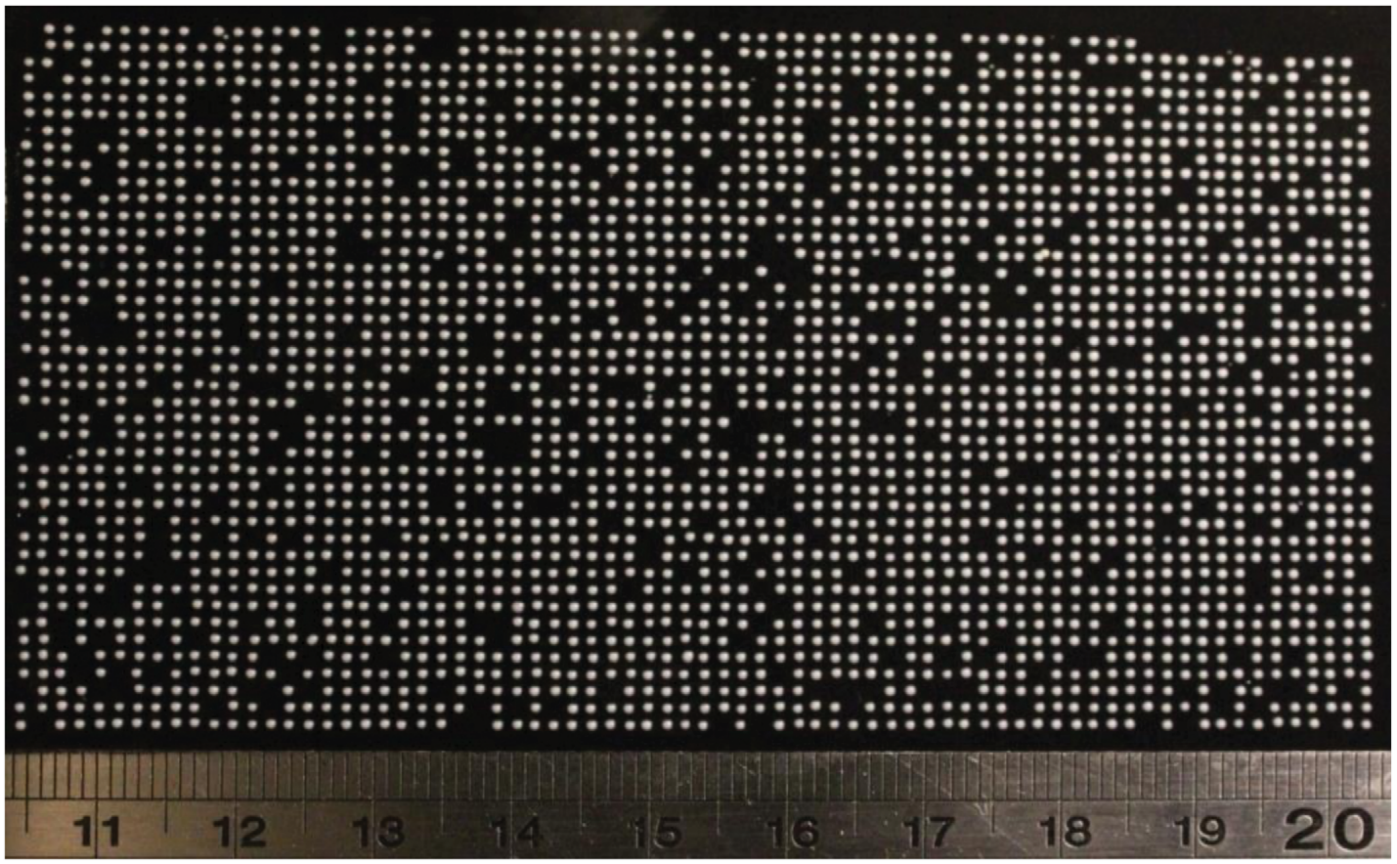
Printing of eukaryotic cells using screening platform. A plate was printed with yeast from the species *Pichia pastoris* in 3066 matrix format onto an YPD agar charcoal plate using the Fluorescence Activated Cell Sorter, showing the proteins carried in eukaryotic cells as well as prokaryotic cells could be put through our screening platform.

We performed a test of this device comparing the time taken to matrix print 4,050 bacteria onto an agar plate using the cell sorter in unmodified form versus fitted with our electronic modification (Fig 5c). To print conventionally, the instrument has to perform 50 y-axis and 81 x-axis translations and under these conditions the time taken to complete a plate was 1563 seconds (SD±6.24 n = 3), we then fitted the sort multiplexer and repeated the experiment. With the multiplexer *in situ*, the instrument performs 50 y-axis translations as before but only 9 x-axis translations and under these conditions, the time taken to complete a plate was 169 seconds (SD±1.53 n = 3). Therefore, when printing with the multiplexer device there was an 89.2% increase in throughput compared to printing with the conventional single stream. Finally, we extended our platform to print eukaryotic cells. Individual yeast cells from the species *Pichia pastoris* were printed in a 3066 matrix (Fig 6).

## Discussion

Our increased understanding of genetic biology has given us the ability to easily manipulate proteins at the genetic level. However, the laboratory screening of protein libraries is mostly limited to classical, laborious microbial techniques unless the protein exhibits markers such as a binding capacity [7] or stable fluorescence [8]. Here we report on a novel, high-throughput screening platform for proteins that cannot be easily screened for on a large scale by existing modalities.

We have shown that using electronic flow sorting, single bacteria, containing a protein of interest, may be deposited in a dense matrix format on solid nutrient media. Individual colonies, arranged in a matrix size up to 15K per plate, may then be rapidly interrogated and characterized using a combination of imaging and bioinformatic approaches. It is worth noting that single bacterial deposition using flow cytometry has been previously reported[24], however the current method extends the practise of single bacterial cell sorting to very high-density format to facilitate mass screening of colonies in a manner that is not constrained to the format of commercial microwell plates. In addition it also provides an integrated and validated screening method. We have then further extended the capabilities of this platform by devising and fabricating an electronic device, that may be easily retrofitted to a commercial fluorescent cell sorter, and which allows a dramatic increase in the rate of cell deposition beyond any currently available technology. Based on the success of this early prototype device, we are in the process of designing a completely self-contained, next-generation electronic sort multiplexer, which will be capable of fulfilling a design criterion of reliably multiplexing 100 sort streams at full sort rate.

We have demonstrated that this system may be used to identify spectral variants of the bioluminescent protein luciferase from the North American Firefly *Photinus pyralis*. The standard microbiological techniques for screening bioluminescent proteins are limited in the number of colonies that can be screened per experiment, as well as lack of control over colony growth and distribution. In contrast, our screening platform has improved upon these techniques by depositing single bacteria in a matrix format; thereby regulating colony growth and distribution, thus refining the accuracy and speed of downstream processing and analysis. We have attempted to explore the limits of manual picking from our bacterial matrices and it is clear that unaided manual picking tends to be less successful at the highest densities tested when compared to lower densities. We expect that an automated colony picker would be a significant addition to this platform, allowing for a higher degree of fidelity; for example the Stringer (Stringer Instruments) can pick colonies at an accuracy of ± 50 microns. The number of colonies that can be qualitatively selected and subsequently screened in a single experiment using our platform exceeds the number that can be screened by current established techniques, such as pin-printing technologies[25] or technologies utilising micro well plates. In addition, our method allows for the interrogation and deposition of single bacterial cells which is not possible using pin-printing techniques. Our analysis pipeline allows for a multipoint emission spectrum to be produced for each resulting individual colony, as well quantifying light intensity at each of these points. This could also be easily extended further to look at for example, the kinetics of light output by each individual colony over time. Bioluminescence is a well-established reporter, which has found many uses in biological research and development, and we believe that our new platform could be easily adapted to use this reporter system in the screening of genetic events and dynamic kinetic reactions.

In this report, we have focused on the use of our screening platform to evaluate bioluminescent targets and in this context, we have utilised flow cytometry to preselect a subset of bacterial candidates for electronic deposition based on their light-scatter, cell cycle phase and fluorescence reporter expression. However, the multi-parametric detection ability of commercial electronic flow sorters coupled with their high-throughput capability (particularly where our sort stream multiplexer is used) implies that this new technique may be used to great effect wherever there is a requirement for library screening. For example, this technique could be extremely useful in the screening of novel complex fluorescent proteins for super-resolution microscopy, including photoactivatable or photoconvertible fluorescent proteins [11, 12]; or may even be used to select out for fluorescent proteins with more desirable properties such as protection from photobleaching. Current techniques available for screening fluorescent proteins [26] tend to use relatively slow, serial interrogation methods and do not therefore scale well to very high throughput unlike the high-speed multiplex deposition and parallel analyses method described here.

Also, the further use of yeast in this screening platform could lead to improvements of standard screening techniques such as the two hybrid system [22]; and points the way towards large scale screening in eukaryotic cells. We believe that the screening platform described here will be the foundation of a new generation of high throughput screening technologies.

## Supporting Information

S1 File Figure A_ Sort multiplexer simplified schematic of device.eps. Figure B_ Sort multiplexer complete schematic.eps. Figure C_ Sort multiplexer PCB foil.eps. Figure D_ Sort multiplexer component layout.eps. Figure E_ Sort multiplexer connection diagram.eps. CODE_Sort multiplexer Arduino system code.docx. VIDEO_Modified MoFlo sorter printing 4050 bacteria using the sort multiplexer.mp4.
(ZIP)

S1 Protocol Detailed protocol for FACS based single colony matrix deposition.docx. (DOCX)
