# Supplementary material for: Flow-Based Single Cell Deposition for High-Throughput Screening of Protein Libraries
Source: PLoS One. 2015 Nov 4;10(11):e0140730. doi: 10.1371/journal.pone.0140730 (PMC4633160; doi:10.1371/journal.pone.0140730)
Supplement: S1 File — Figure A_ Sort multiplexer simplified schematic of device.eps. Figure B_ Sort multiplexer complete schematic.eps. Figure C_ Sort multiplexer PCB foil.eps. Figure D_ Sort multiplexer component layout.eps. Figure E_ Sort multiplexer connection diagram.eps. CODE_Sort multiplexer Arduino system code.docx. VIDEO_Modified MoFlo sorter printing 4050 bacteria using the sort multiplexer.mp4. (ZIP) [file pone.0140730.s001.zip › S2. CODE_Sort multiplexer Arduino system code.docx]

**S2.f: Arduino system code**

*/* eXcytia system code 5.1*

*Arduino DUE Branch*

*2703201501_WORKING*

*Arnold R Pizzey 28^th^ March 2015*

*This code sends a series of n words (Defined by the LIMIT value) to the Attenuator array controlled by a hardware trigger on pin 21*

**/*

volatile **int** **x** = 0; *// Initialise WORD Array counter -note this variable is marked as volatile so that the interrupt routine can reliably incremented it*

**const int** **LIMIT** = 9; // Define the number of sort streams. Sets upper limit of array and define the modulus in the INCREMENT routine

**const int INTERRUPTNUMBER** = 21; // The hardware interrupt to use - pin 21 on the Arduino DUE

/* Define the WORD array

The WORD array contains the attenuation values applied to successive sort pulses

*/

// int WORD[LIMIT] = {0,63,0,63,0,63,0,63,0}; // Used for setting stream position –Uncomment to use

**int WORD**[**LIMIT]** = {0, 4, 8, 13, 18, 24, 35, 46, 63}; //Nine-drop Array –Comment this line when using the stream position setup code on the line above

**void setup**()

{

  f**or** (**int** **Pin** = 34; **Pin** < 41; **Pin**++) //Loop to initialise pins 34-41 as outputs

  { **pinMode**(**Pin**, OUTPUT); //Initialise Pin as output

{ **pinMode**(**INTERRUPTNUMBER**, INPUT); //Set pin as input

  }

}

**attachInterrupt**(**INTERRUPTNUMBER**, **INCREMENT**, FALLING); // Define the interrupt routine (INCREMENT) that will be called on a Falling sort pulse voltage on pin 21

}

**void loop**()

{

  REG_PIOC_ODSR = **WORD**[x] << 2; //Write a 7-bit word from the WORD array to Arduino DUE pins 34-41, this word is send to the eXcytia board and defines the degree of attenuation applied to the next sort pulse -note bitshift operator to shift word 2 places to left

}

**void INCREMENT**() // The interrupt routine called on a Falling sort pulse voltage on pin 21

{

**x** = ++**x** % **LIMIT**; //Increment x by 1 (modulus LIMIT}

}
