# Supplementary material for: Flow-Based Single Cell Deposition for High-Throughput Screening of Protein Libraries
Source: PLoS One. 2015 Nov 4;10(11):e0140730. doi: 10.1371/journal.pone.0140730 (PMC4633160; doi:10.1371/journal.pone.0140730)
Supplement: S1 Protocol — (DOCX) [file pone.0140730.s002.docx]

**S1: Detailed protocol for FACS based single colony matrix deposition**

**Bacterial screening vector**

The bacterial expression plasmid pGex-6p-2 was engineered to be our screening vector. A silent mutation was introduced to remove the MluI site in LacZ (**5’-cgcagcgagtcagtgagcgaggaag-3’; 5’- actgacccgttgcgcgagaagattgtgcac-3’; 5’-tcttctcgcgcaacgggtcagtgggctgatcattaactatccgctggatg-3’; 5’-aaataattcgcgtctggccttcctgtag-3’**) and cloned back in using BstAPI/Bsu36i restriction sites. The GST tag was replaced with the fluorescent protein eGFP (**5’- aacagtattcatggtgagcaagggcgaggagctgttc-3’; 5’- gcgtcttctaaaccatggaaccgccaccgctcttgtacagctcgtccatgccgagagtgatc-3’**). The chromoprotein cjBlue was inserted in frame with eGFP after a SGGGS linker and flanked by the universal restriction sites NcoI/MluI for easy replacement by protein to be screened. All cloning used Phusion^®^ High-Fidelity DNA Polymerase with 5x Phusion HF buffer (*New England Biolabs*), restriction enzymes and buffers from *New England Biolabs* and Quick ligation™ kit (*New England Biolabs*).

**Bacterial culture preparation**

High competency *E.coli* (strain C2523, *New England Biolabs*) were heat shock transformed with bacterial screening vector. After transformation bacteria were expanded in 10ml LB media (*Fisher Chemical*) containing 100µg/ml Carbenicillin (Bioline) in a bacterial Incubator shaker at 37°C overnight. After overnight incubation, bacterial cultures were supplemented with 1mM IPTG (*Fisher Scientific*) and 5ml fresh LB medium with Carbenicillin. The cultures were incubated for a further 3-4 hours, the bacteria reached an OD600 0.6≥0.2. 1.5ml of bacteria were pelleted and then resuspended in 1x Phosphate Buffered Saline, without Calcium/Magnesium (*gibco^®^Life Technologies*) and incubated for 15 minutes at 37°C with the DNA binding dye Hoechst 34580 (*Sigma*) at a final concentration of 4.5µg/ml. Bacteria were pelleted and washed three times with 1xPBS, before been diluted 1:300 in PBS for FACS analysis and printing.

**FACS analysis and printing of single bacteria**

The bacterial suspension was processed on a MoFlo™ XDP Electronic Cell Sorter (*Beckman Coulter*) at >1000<5000 events/seconds. Viable bacteria were identified by the following gating strategy: a primary gate was applied to the Hoechst 34580 histogram and delineated a population of events that had a low DNA staining intensity. The Hoechst gate was carried over to a Forward v Side scatter plot and a further gate was applied to a population displaying a low side-scatter profile, events satisfying these criteria were gated onto a histogram of Forward scatter pulse width and events with low pulse width (singlets) were used as sort candidates. Finally, those bacteria that had been transformed with the screening vector were identified by gating on their eGFP expression.

Bacteria were sorted in desired matrix format directly onto Nunc^™^ OmniTray™ (*Thermo Scientific*) filled with LB agar (*Melford Laboratories Ltd*) containing 4% activated charcoal (*Sigma*) and 100µg/ml Carbenicillin. Plates were incubated at 37°C until discreet colonies were visible. Once colonies had sufficiently developed, plates were sprayed with 10mM IPTG using a commercial airbrush on medium setting (*SP30 Gravity Feed Airbrush SVHC*). Plates were left at room temperature for a further 3-4 hours before analysis.

**Single cell validation**

pGex-6p-2.eGFP and pGex-6p-2.mCherry were transformed into bacteria and samples were prepared for FACS analysis and printing as described above in *Bacterial culture preparation*. The two samples were mixed in an approximate 1:1 ratio and run through the electronic sorter. By referring to the eGFP verses mCherry scatter plot obtained, the sample ratio was adjusted to ensure a 1:1 population of eGFP expressing bacteria to mCherry expressing bacteria. Gating just off the bacterial population and Hoechst 34580 bacteria were printed in a 3,750 matrix format. Plates were then treated as detailed in *FACS analysis and printing of single bacteria.*

Plates were then imaged on the PhotonIMAGER™Optima (*BioSpace Lab*). Two fluorescent images were acquired with a 50mm lens f/1.2, one to detect eGFP expression (440nm_ex_/510-560nm_em_) and the other to detect mCherry expression (540nm_ex_/590-640nm_em_). Images were exported as 8-bit PNG files from the Biospace M3 vision software. A composite image was created by overlaying the eGFP image and mCherry images using the freeware application Image J. The composite images were then analysed using CellProfiler.

**Bioluminescent imaging**

Both x5_FLuc and x5_FLuc red had the required NcoI/MluI restriction sites inserted at the 5’ and 3’ end of the FLuc sequence using the forward and reverse primers (**5’- ctagactgccaaccatggaggacgccaagaacatcaag-3’** and **5’- ctgccctcacgcgtctcttacacggcgatcttgccgcccttc-3’**). Bacterial suspensions were prepared and printed as described in *Bacterial culture preparation* and *FACS analysis and printing of single bacteria*. One plate was printed in 3,750 matrix format using a sample of x5_FLuc and a sample of x5_FLuc_red (first 25 rows x5_FLuc, last 25 rows x5_FLuc_red). Another plate was printed in 3,750 matrix format from a sample of x5_FLuc and x5_FLuc_ red in a ratio of 1:0.005.

Bioluminescent images were acquired on the PhotonIMAGER™Optima with a 50mm, f/1.2 lens. Plates were sprayed with 1mM D-Luciferin (*Regis Technologies*) using an airbrush prior to image acquisition. A lag time of two minutes was used to ensure that the light emission had stabilised before the plates were imaged. A total of six bioluminescent images were acquired using the filters (510-560nm, 550-600nm, 590-640nm, 630-680nm, 670-720nm, 700-750nm, 10 second exposures) starting with the longest wavelength band-pass filter. Images were then saved as greyscale 8-bit PNG files before been analysed.

**Image analysis**

Image data were analyzed using the free software tool CellProfiler. The analysis pipeline consisted of 2 Input modules and 10 analysis modules. Input Modules were ‘Images’ which takes a list of spectral images to be analysed, and ‘Names and types’ which takes the image file list and assigns consistent names to each image file based on keyword recognition for subsequent processing. Pipeline analysis modules are as follows;

- ***Image Math -*** Takes the images identified by the Names And Types module as its input and returns the element-wise maximum value at each pixel location, the output image is assigned the internal name: ***MAXIMISED_IMAGE_STACK***
- ***Apply Threshold* -** Takes the ***MAXIMISED_IMAGE_STACK*** image as its input and applies the Maximum correlation thresholding (MCT) algorithm^1^. Pixel intensities below the threshold are set to zero, those above are maximized, and the resulting binary image is used to mask bioluminescent targets.
- ***Measure Objects Intensity -*** This module is applied to individual spectral images in an analyses set. The module uses the binary image generated by the ***Apply Threshold*** module to mask background regions. Non-masked regions of each image are processed through a number of sizes, morphologic and intensity-based filters to identify objects (colonies) for measurement by the subsequent ***Measure Object Intensity*** module.
- ***Measure Object Intensity* -** This module measures the median intensity of the objects identified in the ***Identify Primary Objects*** module.
- ***Export To Spreadsheet* -** This module allows the export of user-defined measurements of the objects identified in the previous modules.

This data was then processed using a MATLAB script. Intensity data for each individual colony for each of the six filters is input, as well as the x/y coordinates for each colony. Next the intensity data for each colony was normalised, and any red shifted colonies identified (maxima = 1 in band pass filters 3-6). The x/y coordinates for each colony were plotted to create a plate map. On the recreated plate map any red shifted colony was highlighted by the MATLAB script, in addition a 6-point spectral plot was produced for each red shifted colony as well as a table of the intensity values calculated by the CellProfiler pipeline.

**Bioluminescent testing of crude bacterial cultures**

Colonies identified as being red-shifted were picked and expanded in 4ml LB with 100mg/ml Carbenicillin at 37°C in a bacterial shaker overnight, as well as a WT x5_FLuc colony. 3-4 hours before analysis 1mM IPTG was added along with 2ml LB media plus Carbenicillin. Bacteria were pelleted and resuspended in 10x TEM buffer (1M Tris-acetate, 20mM EDTA and 100mM MgSO_4_ at pH 7.8), 5mM ATP (*VWR International*) and 1% Triton-X (*Promega*). 200µl of each sample was added to a black 96 well plate (*Thermo Scientific*) in triplicate. The Thermo Varioskan^™^ Flash multimode reader was used to take a bioluminescent spectrum of each sample. First a kinetic loop was used to take 3 background luminetric readings of each well (1000ms readings, automatic detection). A well loop was then used to first dispense 1mM Luciferin into the well followed by taking a luminetric spectrum (between 500-750nm, every 10nm, 1000ms readings, automatic detection).

**Yeast preparation and printing**

Yeast species *Pichia pastoris* containing the pPICZα (*Invitrogen*) were grown in YPD media with 100mg/ml Zeocin (*Invitrogen*) at 30°C overnight. Yeast were then pelleted and resuspended in 1x PBS and incubated for 15 minutes at 37°C with the DNA binding dye Hoechst 34580 at a final concentration of 4.5µg/ml. Yeast pellets were washed 3 times and diluted in 1x PBS for FACS analysis and printing. Yeast cells were identified on forward scatter/side scatter, and viable yeast cells for printing were gated on the Hoechst 34580 profile. Single yeast cells were deposited in a 3066 matrix format onto a 4% charcoal YPD agar plate with 100mg/ml Zeocin. Plates were grown for 2 days at 30°C.

**References**

1. Padmanabhan, K., Eddy, W. F. & Crowley, J. C. A novel algorithm for optimal image thresholding of biological data. *J. Neurosci. Methods* **193,** 380–384 (2010).
